# Gramian angular field fusion with dual-branch ConvNeXt and channel-aware transformer for vibration sensor-based fault diagnosis

**DOI:** 10.1371/journal.pone.0354912

**Published:** 2026-08-03

**Authors:** Pengjuan Liu, Jindou Ma

**Affiliations:** 1 School of Physics and Electronic-Electrical Engineering, Aba Teachers College, Wenchuan, China; 2 Institute of Modern Physics, Chinese Academy of Sciences, Lanzhou, China; Zhejiang University, CHINA

## Abstract

To address insufficient fault feature representation in vibration sensing signals of rotating machinery, the limited ability of a single image-based representation to characterize complex dynamic information, and the restricted capability of deep models in selecting key features, a fault diagnosis method integrating Gramian Angular Field, dual-branch ConvNeXt, and a channel-aware Transformer is proposed. First, one-dimensional vibration signals are mapped into two types of two-dimensional image representations, namely Gramian Angular Summation Field (GASF) and Gramian Angular Difference Field (GADF), to enhance the structured representation of raw sensing signals from the perspectives of global structural correlation and local dynamic variation. Then, a dual-branch ConvNeXt feature extraction network is constructed to separately model GASF and GADF images, thereby fully exploiting the complementary fault information embedded in the two image representations. On this basis, a channel-aware Transformer module is designed to adaptively enhance fault-sensitive features and suppress redundant information through a channel recalibration mechanism. Meanwhile, the self-attention mechanism is used to capture global dependencies among the fused features. Finally, experiments were conducted on the Paderborn University bearing dataset and the University of Connecticut gear dataset. The results showed that the proposed method achieved accuracies of 97.08% and 98.15% on the two datasets, respectively. It also outperformed the comparison methods in terms of precision, recall, and F1-score, verifying its effectiveness and generalization capability for fault diagnosis of vibration sensing signals.

## Introduction

Rotating machinery is a key component of modern industrial systems and is widely used in energy, manufacturing, transportation, and other critical fields. Its operating condition is directly related to the safety and reliability of industrial systems. Once faults occur in core components such as bearings and gears, equipment performance may be degraded, and severe economic losses or even safety accidents may be caused. Therefore, condition monitoring and fault diagnosis of rotating machinery based on vibration sensing signals are of significant theoretical importance and practical engineering value [1–3].

Existing fault diagnosis methods can generally be divided into mechanism-driven methods and data-driven methods. Mechanism-driven methods rely on physical models and dynamic mechanisms of systems, and fault identification is achieved by establishing accurate mathematical models. However, rotating machinery systems are usually characterized by strong nonlinearity, complex coupling, variable operating conditions, and noise interference. These characteristics make high-precision physical modeling difficult and limit the application of such methods in practical industrial scenarios [4]. In contrast, data-driven methods can directly extract fault-related features from sensor monitoring data without relying on accurate physical models. Therefore, they have received extensive attention in intelligent fault diagnosis of rotating machinery.

Among data-driven methods, traditional machine learning approaches usually depend on manually designed time-domain, frequency-domain, or time-frequency-domain features, which are combined with classifiers such as support vector machines and random forests for fault identification. Although these methods have a certain degree of interpretability, their diagnostic performance largely depends on expert experience and the quality of feature engineering. When vibration sensing signals are affected by nonstationarity, weak impact features, operating condition variations, or background noise, manually extracted features often fail to sufficiently represent subtle differences among different fault states, thereby reducing the generalization ability and diagnostic stability of the model [5].

In recent years, deep learning methods have shown strong feature learning capability in rotating machinery fault diagnosis because deep features can be automatically extracted through multilayer nonlinear mapping [6–8]. As industrial equipment operates under increasingly complex conditions, the adaptability of fault diagnosis models to unknown operating conditions, cross-device scenarios, and small-sample settings has attracted growing attention. Cross-domain diagnosis and domain adaptation methods have been developed to alleviate performance degradation caused by distribution discrepancies between training and testing data. For example, a cross-domain differential attention network (CDDAN) was proposed to enhance the adaptation and transfer of fault features across different operating conditions or domains by introducing a differential attention mechanism [9]. Digital twin-assisted fault diagnosis has also become an emerging research direction. A digital twin-assisted graph contrastive domain adaptation (GCA) network was developed by integrating digital twin sample generation, graph-structured modeling, and contrastive domain adaptation learning, thereby improving feature representation in small-sample and cross-domain diagnosis scenarios [10]. These studies indicate that effective fault feature representation and adaptability to complex operating conditions remain critical issues in intelligent fault diagnosis of rotating machinery. In addition, convolutional neural network (CNN) has been widely used in vibration signal analysis because of their strong local feature extraction ability [11]. However, when one-dimensional vibration time series are directly modeled, the potential structural correlation information contained in the signals is often difficult to fully exploit. To enhance the structured representation ability of one-dimensional sensing signals, time series have been transformed into two-dimensional image representations. Among these methods, Gramian Angular Field (GAF) encodes time series into image forms through polar coordinate mapping, which can preserve temporal dependencies and global structural information to some extent. Thus, effective inputs are provided for deep vision models to extract fault features [12].

Fault diagnosis methods based on GAF representations have made considerable progress, but two limitations remain. First, most existing studies use a single GAF representation or a simple fusion strategy, which does not fully exploit the complementary information between the Gramian Angular SummationField (GASF) and the Gramian Angular Difference Field (GADF). GASF mainly characterizes the global trend and structural correlation of a time series, whereas GADF is more sensitive to local variation patterns and dynamic differences. Therefore, the use of a single representation may lead to insufficient fault information characterization. Second, conventional CNN mainly rely on local convolution operations for feature learning. Although local textures and spatial patterns can be effectively captured, long-range dependencies and global structural correlations are still difficult to model adequately [13].

To compensate for the limitations of CNN in global modeling, Transformer models have recently been introduced into the field of fault diagnosis. Based on the self-attention mechanism, Transformer models can directly model global dependencies among features and have shown good performance in complex temporal modeling and image feature analysis tasks [14]. However, most existing Transformer-based methods mainly focus on dependency modeling along the temporal dimension, spatial dimension, or among patches, while insufficient attention is paid to the importance differences among different channels in fused features. After the fusion of dual-view image features, the fault-sensitive information contained in different channels is usually unbalanced. Some channels may contain critical fault responses, whereas others may introduce redundant information or noise interference. Without an effective channel selection mechanism, the ability of the model to distinguish complex fault patterns may be weakened.

To address these issues, a vibration-sensor-based fault diagnosis method integrating GAF, dual-branch ConvNeXt, and a channel-aware Transformer (CTransformer) is proposed. Unlike a simple cascade of multiple deep learning modules, the core idea of this study is to build a hierarchical and collaborative modeling mechanism for vibration fault feature representation. First, one-dimensional vibration signals are transformed into two image representations, namely GASF and GADF, to enhance the structured representation of the original signals from the perspectives of global structural correlations and local dynamic differences. Then, a dual-branch ConvNeXt is used to perform decoupled feature extraction from the two heterogeneous image representations. This design mitigates feature confusion caused by premature fusion and fully exploits the complementary fault information contained in GASF and GADF. On this basis, a CTransformer is introduced to perform channel reweighting and global dependency modeling on the fused features. Fault-sensitive channels are adaptively enhanced, while redundant information is suppressed, thereby improving the discriminative capability of the model for complex fault patterns.

The main contributions of this paper are summarized as follows:

1. A dual-view representation strategy based on GASF and GADF is proposed for vibration sensing signals. Unlike methods that use a single GAF representation or simple image concatenation, the proposed strategy exploits GASF to characterize global structural correlations and GADF to capture local dynamic differences, thereby providing complementary structural information for fault feature learning.
2. A dual-branch ConvNeXt-based decoupled feature extraction framework is constructed. In this framework, GASF and GADF images are modeled independently to avoid feature confusion caused by directly feeding heterogeneous representations into a single network. As a result, global structural features and local dynamic features can be learned separately, which improves the completeness and discriminability of fault feature representation.
3. A CTransformer fusion module is designed. In this module, channel reweighting is first performed on the fused features, and self-attention is then used to model global dependencies. Thus, fault-sensitive channel enhancement, redundant information suppression, and global feature interaction modeling are achieved simultaneously.

The remainder of this paper is organized as follows. The Related Work section reviews existing studies related to the proposed method. The Proposed Method section describes the overall framework and key modules. The Experimental Results and Analysis section presents the experimental settings, comparative results, ablation studies, and interpretability analysis. Finally, the Conclusion section summarizes the main findings and discusses future work.

## Related work

### Fault diagnosis methods based on image representations and CNN

CNNs have achieved remarkable success in image processing and pattern recognition because of their strong local feature extraction capability, and have been widely applied to rotating machinery fault diagnosis tasks [15,16]. By transforming vibration signals into time-frequency maps or image-based representations such as GAF, CNNs can automatically learn discriminative feature representations, thereby reducing the dependence of traditional methods on manually designed features [17,18].

Previous studies have shown that CNN-based models can achieve high diagnostic accuracy in bearing and gear fault identification. For example, Zhao et al. [19] proposed a CNN model based on multi-information fusion, which effectively enhanced fault feature representation. Ruan et al. [20] improved the ability of the model to capture fault frequency characteristics under different conditions by optimizing the design of convolution kernel parameters. In addition, lightweight or deep CNN architectures, such as ResNet and MobileNet, have also been introduced into fault diagnosis tasks to reduce computational complexity while maintaining diagnostic accuracy [21,22].

However, CNN essentially rely on local convolution operations, and their receptive fields are limited by the kernel size and network depth. Therefore, long-range dependencies are difficult to model effectively. Moreover, traditional CNN architectures usually adopt fixed hierarchical designs, which limits their adaptability to multi-scale dynamic features in complex industrial data. These problems are particularly prominent in the analysis of nonstationary and strongly coupled vibration signals, thereby restricting further improvements in model performance.

### Transformer-based fault diagnosis methods

Transformer was first proposed by Vaswani et al. [23] and has achieved breakthrough progress in natural language processing. Its core component is the self-attention mechanism, which can directly model dependencies between any positions in a sequence. Therefore, Transformer has significant advantages in global feature modeling. In recent years, Transformer has gradually been introduced into time-series analysis and fault diagnosis, showing promising application potential [24,25].

In rotating machinery fault diagnosis, Transformer is usually combined with CNN or other feature extraction methods to integrate local feature learning with global dependency modeling. For example, the Anomaly Transformer proposed by Xu et al. [26] improved anomaly detection capability through an association discrepancy mechanism. In some studies, extracted features have been fed into Transformer modules to further model complex patterns [27]. In addition, improved models incorporating attention mechanisms have also been used to enhance the focus of models on key features [28].

Although Transformer has advantages in global modeling, several limitations still remain in existing studies. On the one hand, most methods mainly focus on dependencies along the temporal or spatial dimension, while importance differences among feature channels are not explicitly modeled. This may introduce redundant information and degrade diagnostic performance. On the other hand, most studies use a single feature input or a simple fusion strategy, and the complementary information among multi-source features is not sufficiently exploited. Consequently, the representation capability of these models for complex fault patterns is limited.

## Proposed method

### Overall architecture

**Fig 1:**
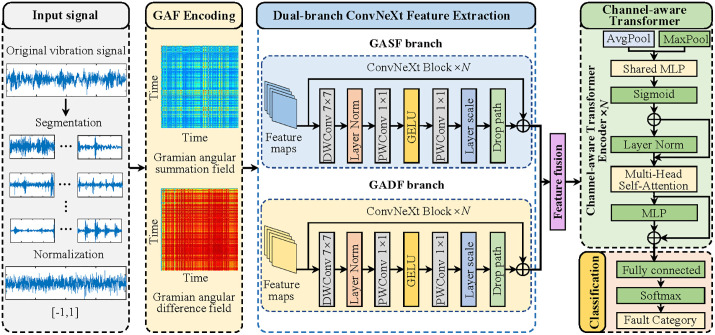
Overall framework of the proposed GAF-ConvNeXt-CTransformer model.

To address the limited feature representation capability of one-dimensional vibration signals and the insufficient utilization of multi-source information by deep models in rotating machinery fault diagnosis, a fault diagnosis framework integrating GAF with a dual-branch ConvNeXt and a CTransformer is proposed. The overall architecture is shown in Fig 1. It should be noted that the proposed framework is not a simple cascade of GAF, ConvNeXt, and Transformer modules. Instead, it is specifically designed around the feature representation requirements of vibration-based fault diagnosis.In this framework, GASF and GADF are used to provide two complementary types of information, namely global structural information and local dynamic information. The dual-branch ConvNeXt is employed to independently model these two heterogeneous representations, thereby reducing premature mixing between different feature spaces. The CTransformer further enhances the fused features from both the channel dimension and the global dependency dimension. In this way, the proposed method forms a continuous optimization process from signal representation and decoupled feature extraction to channel-aware global modeling, which constitutes the main methodological innovation of this study.

First, in the signal preprocessing stage, the raw one-dimensional vibration signals are segmented using a fixed window to construct the sample set. Meanwhile, normalization is performed to map the data into the range of [−1, 1], thereby eliminating scale differences and improving the stability of model training. This process provides a unified data scale for subsequent image-based modeling while preserving the original signal information as much as possible.

Then, the GAF method is used to transform one-dimensional time series into two-dimensional image representations, namely GASF and GADF. Specifically, GASF mainly reflects the overall trend and global correlation of the time series, whereas GADF focuses on local variation relationships and dynamic difference features. By constructing dual-view image representations, the raw signals can be represented from different structural spaces, thereby enhancing the representation capability of the data.

In the feature extraction stage, a dual-branch ConvNeXt network is constructed to model GASF and GADF images in parallel. The key idea of this design is to decouple two types of image representations with different structural characteristics, enabling the model to learn global structural information and local variation features separately. Compared with a single-branch structure, the dual-branch architecture can effectively exploit the complementary relationship between the two representations, thus improving the completeness and expressiveness of the extracted features. In addition, ConvNeXt enhances feature learning capability while maintaining computational efficiency by introducing large convolution kernels and normalization strategies. On this basis, the feature fusion module integrates the features extracted from the two branches. This process not only preserves key information from different branches but also enables collaborative representation of multi-source features, providing richer inputs for subsequent global modeling.

Finally, in the classification stage, a CTransformer module is introduced to further model the fused features. Based on the conventional Transformer structure, this module incorporates a channel attention mechanism. By explicitly modeling the importance distribution of feature channels, fault-related key features are adaptively enhanced, and the interference of redundant information is suppressed. Meanwhile, the self-attention mechanism is used to capture global dependencies among features, thereby enabling efficient representation and accurate classification of complex fault patterns.

### Gramian Angular Field

GAF [29] is an image-based representation method that maps a one-dimensional time series into a two-dimensional image. Its core idea is to encode the amplitude information of a sequence through polar coordinate transformation, thereby characterizing the structural features of the time series in a two-dimensional space. This method can preserve temporal dependencies while converting the raw sequence into an image representation suitable for visual feature modeling, thus providing richer structural information for subsequent feature extraction.

Given a time series $X=\left\{x_{\text{1}},x_{\text{2}},\cdots ,x_{n}\right\}$, *X* is first normalized as follows:


$$\begin{array}{c} {\tilde{x}}_{i}=\frac{\left(x_{i}-max\left(X\right)\right)+\left(x_{i}-min\left(X\right)\right)}{max\left(X\right)-min\left(X\right)} \end{array}$$ (1)

Then, the normalized sequence is mapped into the polar coordinate system:


$$\begin{array}{c} \left\{ \begin{array}{c} \phi_{i}=arccos\left({\tilde{x}}_{i}\right)\;,-1\leq {\tilde{x}}_{i}\leq 1,{\tilde{x}}_{i}\in \tilde{X}\\ r_{i}=\frac{i}{n},i=1,2,\cdots ,n \end{array}\right. \end{array}$$ (2)

where $\phi_{i}$ denotes the polar angle of the *i*-th sampling point, and $r_{i}$ denotes its radius.

On this basis, the two-dimensional representation of the time series can be obtained by constructing trigonometric relationships between different time points. Specifically, GAF mainly includes two forms: GASF and GADF. GASF describes the variation relationship of the time series through the cosine of the angle summation, which is defined as


$$\begin{array}{c} G_{s}=\left[\begin{array}{ccc} cos(\phi_{\text{1}}+\phi_{\text{1}}) & cos(\phi_{\text{1}}+\phi_{\text{2}}) & \cdots \\ cos(\phi_{\text{2}}+\phi_{\text{1}}) & cos(\phi_{\text{2}}+\phi_{\text{2}}) & \cdots \\ \vdots & \vdots & \vdots \\ cos(\phi_{n}+\phi_{\text{1}}) & cos(\phi_{n}+\phi_{\text{2}}) & \cdots \end{array}\begin{array}{c} cos(\phi_{\text{1}}+\phi_{n})\\ cos(\phi_{\text{2}}+\phi_{n})\\ \vdots \\ cos(\phi_{n}+\phi_{n}) \end{array}\right] \end{array}$$ (3)

where $\varphi_{i}(i=1,2,\cdots ,n)$ represent the polar angles of two time points.

GADF describes the variation relationship of the time series through the sine of the angular difference, which is defined as


$$\begin{array}{c} G_{d}=\left[\begin{array}{ccc} sin(\phi_{\text{1}}-\phi_{\text{1}}) & sin(\phi_{\text{1}}-\phi_{\text{2}}) & \cdots \\ sin(\phi_{\text{2}}-\phi_{\text{1}}) & sin(\phi_{\text{2}}-\phi_{\text{2}}) & \cdots \\ \vdots & \vdots & \vdots \\ sin(\phi_{n}-\phi_{\text{1}}) & sin(\phi_{n}-\phi_{\text{2}}) & \cdots \end{array}\begin{array}{c} sin(\phi_{\text{1}}-\phi_{n})\\ sin(\phi_{\text{2}}-\phi_{n})\\ \vdots \\ sin(\phi_{n}-\phi_{n}) \end{array}\right] \end{array}$$ (4)

**Fig 2:**
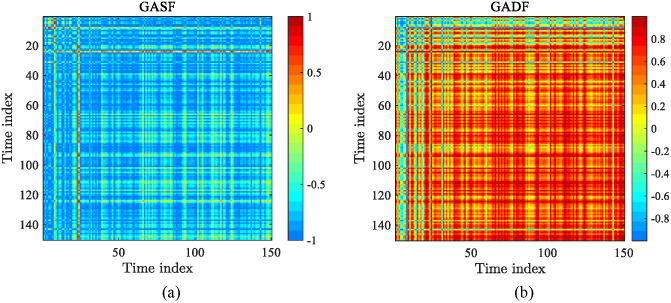
Illustration of GASF and GADF representations: (a) GASF image; (b) GADF image.

By simultaneously constructing GASF and GADF images, the original vibration signal can be modeled from two perspectives: global structure and local dynamics. GASF is more effective in capturing global structural correlations, whereas GADF is more sensitive to local dynamic variations. Fig 2 presents the GASF and GADF representations of the same vibration signal. It can be observed that the two representations exhibit distinct structural characteristics, indicating that they can provide complementary information for subsequent fault feature extraction.

### Dual-branch ConvNeXt feature extraction module

ConvNeXt integrates the advantages of traditional CNN and Transformer-inspired design principles, thereby improving feature representation while maintaining computational efficiency. A ConvNeXt block is mainly composed of depthwise convolution, layer normalization, pointwise convolution, and residual connection. Its basic computation can be formulated as


$$\begin{array}{c} Y=X+PWConv_{\text{2}}\left(GELU\left(PWConv_{\text{1}}\left(LN\left(DWConv\left(X\right)\right)\right)\right)\right) \end{array}$$ (5)

where PWConv(·) denotes pointwise convolution, DWConv(·) denotes depthwise convolution, and LN(·) denotes layer normalization.

Specifically, ConvNeXt enlarges the effective receptive field by introducing a large convolution kernel, such as 7 × 7, thereby enhancing the ability to capture global spatial information. In addition, Layer Normalization and GELU activation are employed to improve training stability and batch consistency. Therefore, ConvNeXt can effectively extract spatial texture features and structural patterns from GAF images.

After GAF encoding, GASF and GADF characterize the structural properties of time series from different perspectives. GASF mainly reflects the overall trend and global correlation of the sequence, whereas GADF is more sensitive to local variations and dynamic differences. If early fusion is directly performed at the input stage, or if the two types of features are simply concatenated along the channel dimension, information from different representation spaces may be mixed prematurely during low-level feature learning, thereby weakening their distinct representational capabilities. To address this issue, a dual-branch ConvNeXt structure is adopted to decouple the modeling of GASF and GADF. The two branches are used to learn global structural features and local dynamic features separately, and feature fusion is then performed in the high-level semantic space. This design helps mitigate feature confusion caused by simple fusion and enables the complementary fault information between the two image representations to be more fully exploited.

Let the GASF and GADF images be denoted as *X*_*GASF*_ and *X*_*GADF*_, respectively. The dual-branch feature extraction process can be expressed as


$$\begin{array}{c} F_{GASF}=f_{ConvNeXt}\left(X_{GASF}\right) \end{array}$$ (6)

$$\begin{array}{c} F_{GADF}=f_{ConvNeXt}\left(X_{GADF}\right) \end{array}$$ (7)

where $f_{ConvNeXt}(\cdot )$ denotes the ConvNeXt feature extraction network.

After dual-branch feature extraction, feature fusion is required to form a unified representation. In this paper, a weighted fusion strategy is used to integrate the two types of features, which is formulated as


$$\begin{array}{c} F=\alpha F_{GASF}+(1-\alpha )F_{GADF} \end{array}$$ (8)

where *α* is the fusion weight. This strategy preserves the structural simplicity of the model while achieving effective integration of multi-source features. The fused representation contains both global trend information and local dynamic characteristics, thereby constructing a more complete feature space.

The dual-branch ConvNeXt feature extraction module not only improves the utilization of multi-source information but also provides discriminative high-dimensional features for the subsequent CTransformer. This design lays a solid foundation for the accurate identification of complex fault patterns.

### Channel-aware transformer

After the dual-branch features of GASF and GADF are fused, the fault-sensitive information contained in different deep feature channels is not evenly distributed. Some feature channels may show strong responses to local impacts, periodic modulation, structural texture variations, or changes in global correlations, whereas other channels may contain redundant information or noise-related responses. Therefore, a CTransformer is introduced after feature fusion.

It should be noted that the “channels” in this study do not refer to the original vibration sensor channels. Instead, they are deep feature channels formed after GASF and GADF images are extracted and fused by the dual-branch ConvNeXt. Each feature channel can be interpreted as a latent fault-related semantic response learned by the model. The channel-aware mechanism adaptively enhances fault-sensitive channels and suppresses redundant responses according to their contributions to fault discrimination, thereby improving the utilization efficiency of the fused features.

Let the fused feature be denoted as $F\in {\mathbb{R}}^{C\times H\times W}$. First, global average pooling and global max pooling are performed along the spatial dimension to extract channel-level statistical information:


$$\begin{array}{c} F_{avg}=AvgPool\left(F\right) \end{array}$$ (9)

$$\begin{array}{c} F_{max}=MaxPool\left(F\right) \end{array}$$ (10)

Then, the two types of statistical features are fed into a shared multilayer perceptron for nonlinear mapping, and a channel weight vector is generated through a sigmoid function:


$$\begin{array}{c} W_{c}=\sigma \left(MLP\left(F_{avg})+MLP(F_{max}\right)\right) \end{array}$$ (11)

where σ denotes the Sigmoid function, and $W_{c}\in {\mathbb{R}}^{C\times 1\times 1}$ represents the channel attention weight vector.

Finally, the channel weights are applied to the original fused feature to achieve channel recalibration:


$$\begin{array}{c} F^{\text{'}}=F⊙W_{c} \end{array}$$ (12)

where ⊙ denotes element-wise multiplication.

After channel recalibration is completed, *F′* is reshaped into a sequence and fed into the Transformer encoder for global dependency modeling. Specifically, the feature dimension is used as the sequence representation, and linear projections are adopted to generate the query, key, and value matrices:


$$\begin{array}{c} Q=F^{\text{'}}W_{Q},K=F^{\text{'}}W_{K},V=F^{\text{'}}W_{V} \end{array}$$ (13)

where $W_{Q},W_{K},W_{V}$ are learnable parameters.

Based on the self-attention mechanism, the correlations among features are calculated as:


$$\begin{array}{c} Attention\left(Q,K,V\right)=\text{Softmax}\left(\frac{QK^{T}}{\sqrt{d_{k}}}\right)V \end{array}$$ (14)

Then, a multi-head attention mechanism is used to capture dependencies among features in different subspaces, thereby enhancing the ability to model complex fault patterns. In addition, residual connections and layer normalization are employed in the Transformer encoder to ensure stable information propagation. The calculation process can be expressed as:


$$\begin{array}{c} Z=LN\left(F^{{\;}^{\prime }}+MHA\left(F^{{\;}^{\prime }}\right)\right) \end{array}$$ (15)

$$\begin{array}{c} F^{{\;}^{\prime \prime }}=LN\left(Z+MLP\left(Z\right)\right) \end{array}$$ (16)

Finally, the global feature representation is fed into a fully connected layer, and the fault category is predicted through the Softmax function:


$$\begin{array}{c} \hat{y}=\text{Softmax}(WF^{\text{''}}+b) \end{array}$$ (17)

where *W* and *b* denote the learnable weight matrix and bias term, respectively.

In summary, CTransformer enables efficient modeling of fused features through a two-stage mechanism that combines channel recalibration and global dependency modeling. The channel attention mechanism selects critical features from the channel dimension and improves feature utilization, whereas the self-attention mechanism captures global dependencies and enhances the modeling capability for complex fault patterns.

## Experiments and results

### Datasets and experimental settings

#### Paderborn university bearing dataset.

The Paderborn University (PU) bearing dataset from Germany [30] is a publicly available benchmark dataset for bearing fault diagnosis. This dataset consists of data collected from six healthy bearings, twelve artificially damaged bearings, and fourteen bearings with real damage generated through accelerated lifetime tests. Therefore, different bearing health states, including normal states, artificial damage states, and real damage states, are covered. The original vibration signals were collected under different operating conditions, including rotational speed, load torque, and radial load. Thus, the dataset contains vibration signals under different bearing damage states and reflects, to some extent, the influence of speed and load variations on vibration signal characteristics.

The detailed information on the selected bearing condition states is listed in Table 1. A total of 1200 samples were selected from the PU dataset, including 12 bearing condition states with 100 samples for each state. Each sample contained 1600 sampling points. Therefore, the window length and stride were both set to 1600, corresponding to an overlap ratio of 0%. The samples were randomly divided into training and testing sets at a ratio of 4:1.

**Table 1:** Bearing condition state categories in the PU dataset.

Label	Bearing code	Bearing condition	Damage location	Number of samples
1	K001	No fault	Normal condition	100
2	K002	No fault	Normal condition	100
3	K003	No fault	Normal condition	100
4	K004	No fault	Normal condition	100
5	KA01	Outer race	Artificial damage	100
6	KA03	Outer race	Artificial damage	100
7	KI01	Inner race	Artificial damage	100
8	KI03	Inner race	Artificial damage	100
9	KA04	Outer race	Real damage	100
10	KA15	Outer race	Real damage	100
11	KI04	Inner race	Real damage	100
12	KI14	Inner race	Real damage	100

To ensure sufficient experimental data and cover different bearing states, including normal, artificial damage, and real damage states, 12 bearing health or damage states were selected from the PU dataset. Specifically, K001-K004 represent different healthy bearing states; KA01, KA03, KA04, and KA15 represent outer-race damage states; and KI01, KI03, KI04, and KI14 represent inner-race damage states. Therefore, the 12 labels used in the PU dataset more accurately represent different bearing health states or damage states, rather than coarse-grained fault types. This setting was adopted to evaluate the ability of the proposed method to identify fine-grained differences among bearing states.

After the training and testing sets were determined, normalization and GASF/GADF image transformation were performed separately within each set.

#### University of Connecticut gear dataset.

The University of Connecticut (UC) gear dataset [31] was collected from a two-stage gearbox with replaceable gears at a sampling frequency of 20 kHz. The detailed definitions of the gear fault categories are listed in Table 2. This dataset contains nine gear condition categories, including healthy condition, missing tooth, root crack, spalling, and five degrees of chipping damage. Each category contains 104 samples, resulting in a total of 936 samples. Each sample consists of 3600 sampling points. Therefore, the window length and stride were both set to 3600, corresponding to an overlap ratio of 0%. The samples were randomly divided into training and testing sets at a ratio of 10:3.

**Table 2:** Gear fault categories in the UC dataset.

Label	Gear condition	Damage location	Number of samples
1	Healthy	None	104
2	Missing tooth	Tooth	104
3	Root crack	Tooth root	104
4	Spalling	Tooth surface	104
5	Chipping tip 5a	Tooth tip	104
6	Chipping tip 4a	Tooth tip	104
7	Chipping tip 3a	Tooth tip	104
8	Chipping tip 2a	Tooth tip	104
9	Chipping tip 1a	Tooth tip	104

Accuracy, macro-averaged Precision, macro-averaged Recall, and macro-averaged F1-score were used as evaluation metrics, as defined in Eqs. (18)-(21). In these equations, *TP*, *TN*, *FP*, and *FN* denote true positives, true negatives, false positives, and false negatives, respectively. For the multiclass classification task, Precision, Recall, and F1-score were calculated using macro averaging. Specifically, the corresponding metric was first calculated for each class and then averaged across all classes, so that the diagnostic performance for different fault categories could be evaluated fairly.


$$\begin{array}{c} Accuracy=\frac{TP+TN}{TP+TN+FP+FN} \end{array}$$ (18)

$$\begin{array}{c} Precision=\frac{TP}{TP+FP} \end{array}$$ (19)

$$\begin{array}{c} Recall=\frac{TP}{TP+FN} \end{array}$$ (20)

$$\begin{array}{c} F1\_Score=\frac{2\times Precision\times Recall}{Precision+Recall} \end{array}$$ (21)

To improve the reproducibility of the proposed method, the main parameter settings for data processing, GAF image transformation, ConvNeXt branches, the CTransformer module, and model training are summarized in Table 3. The same data processing procedure and training parameters were used in all comparative and ablation experiments to ensure fairness and comparability. Accuracy, macro-averaged Precision, macro-averaged Recall, and macro-averaged F1-score were used as evaluation metrics.

**Table 3:** Main parameter settings of the proposed method.

Parameter category	Parameter category	Parameter category	Parameter category	Parameter category	Parameter category
Data processing	Overlap ratio	0%	CTransformer	Number of attention heads	4
GAF image	Image size	227 × 227 × 3	CTransformer	Number of self-attention layers	2
ConvNeXt	Number of branches	2	CTransformer	Key channels	256
ConvNeXt	Branch parameters	Not shared	Training parameter	Optimizer	Adam
ConvNeXt	Stage channels	64,128,256	Training parameter	Epochs	100
ConvNeXt	Depthwise kernel size	7 × 7	Training parameter	Batch size	64
CTransformer	Channel weight dimension	256	Training parameter	Learning rate	0.0001

## Discussion

**Fig 3:**
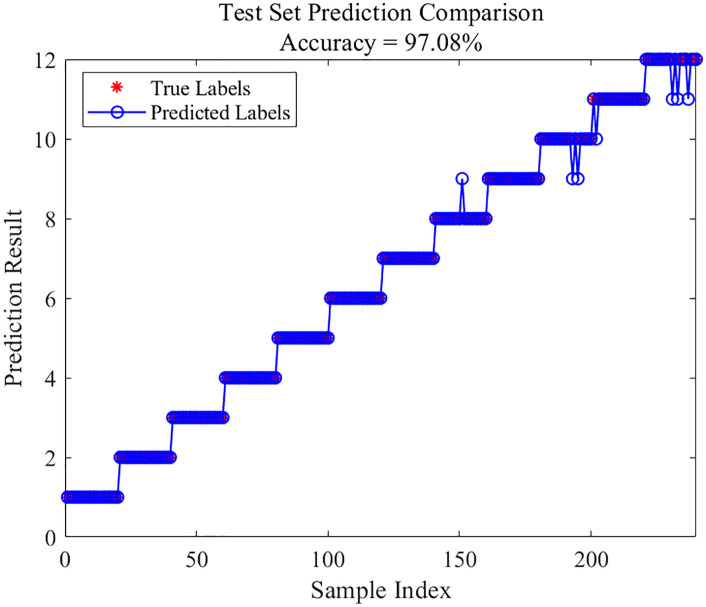
Test set prediction comparison on the PU dataset.

**Fig 4:**
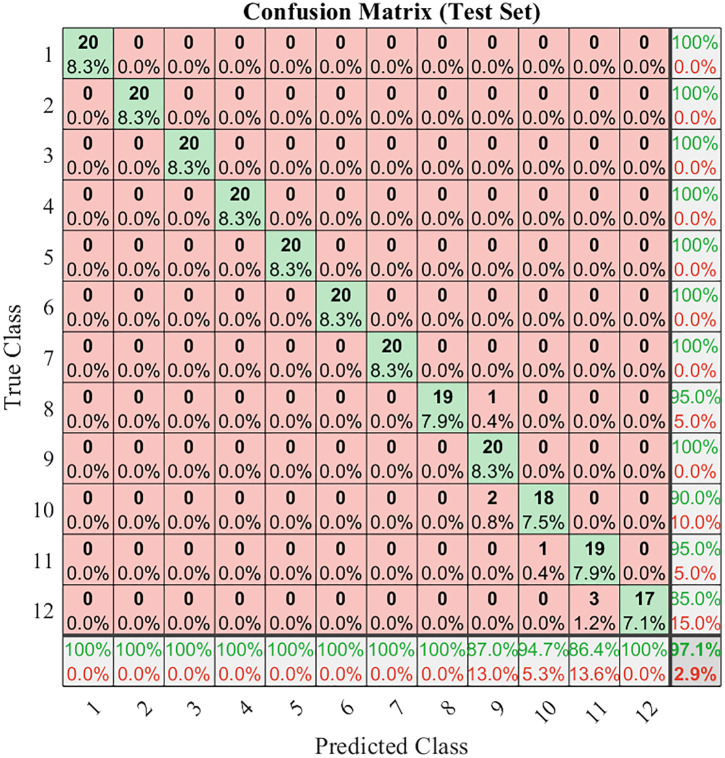
Confusion matrix on the PU dataset (test set).

To evaluate the effectiveness of the proposed method in the PU bearing health-state identification task, the prediction results and confusion matrix of the test set were analyzed, as shown in Figs 3 and 4.

As shown in Fig 3, the predicted labels are highly consistent with the true labels, and most test samples are correctly assigned to their corresponding classes. Only a few deviations are observed, mainly between adjacent bearing health states, indicating that certain bearing states still have similar feature distributions. Overall, the predicted curve almost overlaps with the true labels, demonstrating that the proposed method can effectively extract discriminative features from different bearing health states.

The confusion matrix in Fig 4 further shows that most classification results are concentrated along the main diagonal, indicating high recognition performance for most classes. Each class in the test set contains 20 samples. Classes 1–7 and class 9 were completely correctly classified, with an accuracy of 100%. A small number of misclassifications occurred mainly in classes 8, 10, 11, and 12. Overall, 233 out of 240 test samples were correctly identified, corresponding to an overall accuracy of 97.08%. These results indicate that the proposed method achieves good classification performance on the PU dataset and can effectively distinguish different bearing health states.

**Fig 5:**
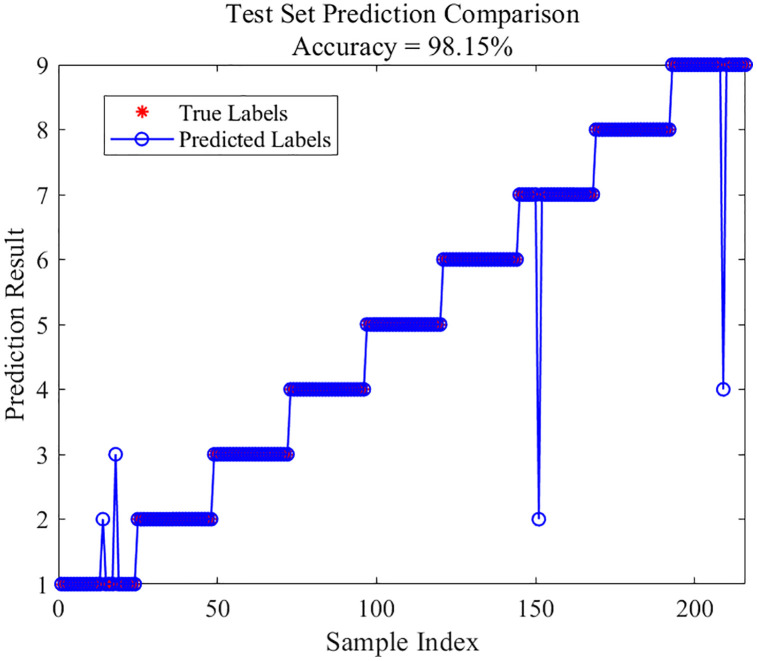
Test set prediction comparison on the UC dataset.

**Fig 6:**
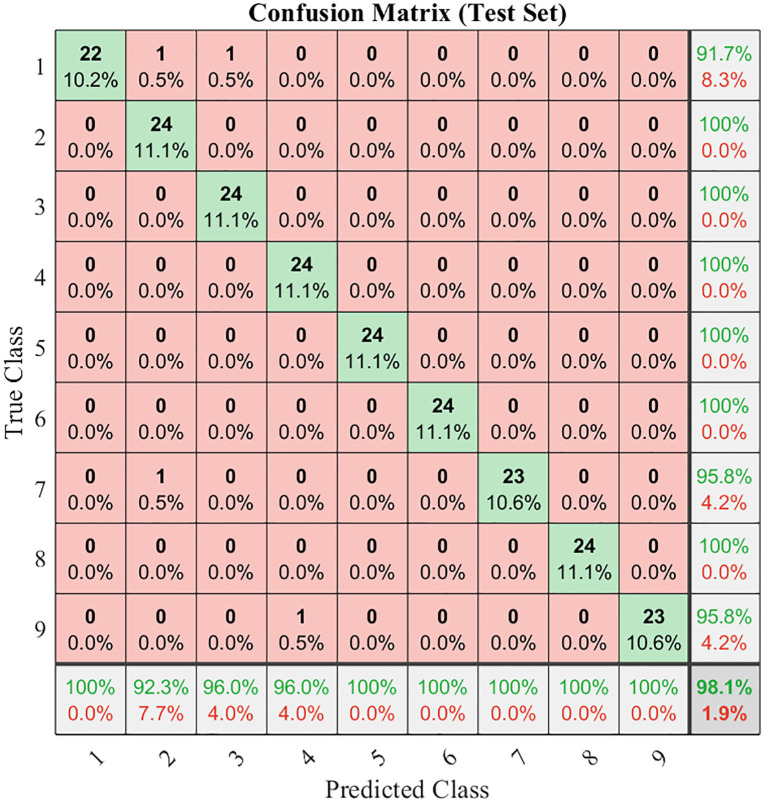
Confusion matrix on the UC dataset (test set).

To further evaluate the applicability of the proposed method to gear fault diagnosis, experiments were conducted on the UC gear dataset. The test results are shown in Figs 5 and 6.

As shown in Fig 5, the predicted labels are highly consistent with the true labels. Most test samples were correctly classified, and only a few prediction deviations were observed. This indicates that the proposed method can effectively learn discriminative features from different gear conditions and maintain good classification stability on the UC dataset.

The confusion matrix in Fig 6 further shows that most classification results are concentrated along the main diagonal, indicating high recognition performance for most gear conditions. Each class in the test set contains 24 samples. Classes 2–6 and class 8 were completely correctly classified, with a recognition accuracy of 100%. A small number of misclassifications occurred mainly in classes 1, 7, and 9. Overall, 212 out of 216 test samples were correctly identified, corresponding to an overall accuracy of 98.15%. These results indicate that the proposed method achieves good fault recognition performance on the UC gear dataset, although slight confusion still exists among some gear conditions with similar features.

### Comparative experiments

To further verify the effectiveness and superiority of the proposed method, several deep learning models were selected for comparison, including CNN, ResNet, ConvNeXt, Transformer, and ViTransformer. These models represent classical convolutional neural networks, residual networks, modern convolutional architectures, and self-attention-based methods, respectively, and can comprehensively reflect the performance of different modeling mechanisms. To ensure the fairness of the comparison, all models were trained under the same data partition, training epochs, and optimization strategy, and their performance was evaluated using the same metrics. The basic settings of the comparison models are as follows.

1. CNN adopts a classical convolutional neural network structure composed of multiple convolutional and pooling layers. The convolution kernel size was set to 3 × 3, and the model was used to extract local features.
2. ResNet introduces residual connections into deep convolutional networks, which alleviates the gradient vanishing problem during training and enhances feature representation capability.
3. ConvNeXt adopts a modern convolutional network architecture, in which large convolution kernels and layer normalization are introduced to improve feature perception and training stability.
4. Transformer adopts the standard Transformer encoder structure and uses the multi-head self-attention mechanism to model global dependencies among features.
5. Vision Transformer divides the input image into multiple patches and models their relationships through the self-attention mechanism, thereby learning global image features.

As shown in Table 4, the proposed method achieved the best results in terms of Accuracy, Precision, Recall, and F1-score over five repeated experiments on the PU dataset. Compared with CNN, ResNet, ConvNeXt, Transformer, and ViTransformer, the proposed method exhibited higher average classification performance. Specifically, the Accuracy reached 97.08%, which was 2.32% higher than that of the second-best ViTransformer. The Precision, Recall, and F1-score reached 97.16%, 96.94%, and 97.05%, with improvements of 2.08%, 3.02%, and 2.55%, respectively. These results indicate that the collaborative use of GASF/GADF dual-view representation, dual-branch ConvNeXt feature extraction, and the CTransformer effectively improves the recognition performance for PU bearing health states.

**Table 4:** Evaluation metrics for various comparative networks (PU).

Model	Accuracy (%)	Precision (%)	Recall (%)	F1-Score (%)
CNN	71.58 ± 1.24	72.24 ± 1.30	70.73 ± 1.27	71.48 ± 1.28
ResNet	82.41 ± 0.93	82.86 ± 0.89	81.37 ± 0.95	82.11 ± 0.92
ConvNeXt	88.19 ± 0.79	87.91 ± 0.81	88.83 ± 0.76	88.37 ± 0.78
Transformer	93.14 ± 0.62	92.87 ± 0.67	93.66 ± 0.60	93.26 ± 0.63
ViTransformer	94.76 ± 0.56	95.08 ± 0.54	93.92 ± 0.58	94.50 ± 0.56
Proposed Method	97.08 ± 0.36	97.16 ± 0.32	96.94 ± 0.39	97.05 ± 0.34

In addition, the proposed method showed small standard deviations across the five repeated experiments. The standard deviations of Accuracy, Precision, Recall, and F1-score were 0.36%, 0.32%, 0.39%, and 0.34%, respectively, indicating good stability under different random splits. The 95% confidence intervals of Accuracy, Precision, Recall, and F1-score were further calculated as [96.63%, 97.53%], [96.76%, 97.56%], [96.46%, 97.42%], and [96.63%, 97.47%], respectively. These narrow confidence intervals further demonstrate the stable diagnostic performance of the proposed method on the PU dataset.

As shown in Table 5, the proposed method achieved the best results in terms of Accuracy, Precision, Recall, and F1-score over five repeated experiments on the UC gear dataset. Specifically, the Accuracy reached 98.15%, while the Precision, Recall, and F1-score reached 98.24%, 98.03%, and 98.13%, respectively. Compared with the second-best ViTransformer, the proposed method improved Accuracy, Precision, Recall, and F1-score by 2.34%, 2.06%, 3.00%, and 2.53%, respectively. These results indicate that the proposed method can extract more discriminative features from UC gear data and achieves better classification performance in gear fault diagnosis.

**Table 5:** Evaluation metrics for various comparative networks (UC).

Model	Accuracy (%)	Precision (%)	Recall (%)	F1-Score (%)
CNN	73.09 ± 1.17	73.58 ± 1.21	72.14 ± 1.18	72.85 ± 1.19
ResNet	84.08 ± 0.86	84.63 ± 0.82	82.94 ± 0.88	83.78 ± 0.85
ConvNeXt	89.61 ± 0.72	89.08 ± 0.75	90.21 ± 0.70	89.64 ± 0.72
Transformer	94.36 ± 0.58	94.11 ± 0.60	94.79 ± 0.55	94.45 ± 0.57
ViTransformer	95.81 ± 0.50	96.18 ± 0.48	95.03 ± 0.53	95.60 ± 0.51
Proposed Method	98.15 ± 0.34	98.24 ± 0.31	98.03 ± 0.37	98.13 ± 0.33

In addition, the proposed method showed small standard deviations across the five repeated experiments. The standard deviations of Accuracy, Precision, Recall, and F1-score were 0.34%, 0.31%, 0.37%, and 0.33%, respectively, indicating good stability under different random splits. The 95% confidence intervals of Accuracy, Precision, Recall, and F1-score were further calculated as [97.73%, 98.57%], [97.86%, 98.62%], [97.57%, 98.49%], and [97.72%, 98.54%], respectively. These narrow confidence intervals further demonstrate the stable diagnostic performance of the proposed method on the UC dataset.

### Ablation study

To verify the effectiveness of GASF, GADF, dual-branch ConvNeXt, and CTransformer, ablation experiments were conducted under the same experimental settings. Model components were gradually removed or replaced to systematically analyze the contribution of each module to the overall performance. To avoid computational redundancy caused by repeated experiments while ensuring the representativeness of the analysis, the PU dataset, which contains more complex operating conditions and richer fault categories, was selected for the ablation study. Accuracy, precision, recall, and F1-score were used as the evaluation metrics.

In the first two experiments, GASF and GADF were used separately as single inputs, and a single-branch ConvNeXt was adopted for feature extraction. In the third experiment, both GASF and GADF were introduced, and a dual-branch ConvNeXt was used for parallel feature modeling. In the fourth experiment, the CTransformer was further incorporated into the dual-branch ConvNeXt, forming the complete model. By progressively adding model components, the contribution of each module to the diagnostic performance could be systematically evaluated.

The ablation results are shown in Table 6. When only a single GAF representation was used, the Accuracy values of the models using GASF and GADF were 93.28% and 94.16%, respectively. This indicates that both GAF representations can provide effective fault-related features, but a single representation is still insufficient to fully characterize complex vibration signals. When GASF and GADF were jointly introduced and modeled using the dual-branch ConvNeXt, the Accuracy increased to 96.42%, and the F1-score reached 96.47%. This demonstrates that the dual-branch structure can effectively integrate the complementary information of the two GAF representations and enhance the feature representation capability of the model. After CTransformer was further introduced, the complete model achieved an Accuracy of 97.08%, with Precision, Recall, and F1-score values of 97.34%, 97.08%, and 97.21%, respectively. These results indicate that CTransformer can strengthen critical features and model global dependencies, thereby further improving fault recognition performance. Overall, each module contributes positively to the performance improvement of the proposed model.

**Table 6:** Ablation experiment results on the PU dataset.

GASF	GADF	Single-branch ConvNeXt	Dual-branch ConvNeXt	CTransformer	Accuracy	Precision	Recall	F1_Score
√		√			93.28%	93.74%	92.81%	93.27%
	√	√			94.16%	94.52%	93.79%	94.15%
√	√		√		96.42%	96.18%	96.77%	96.47%
√	√		√	√	97.08%	97.34%	97.08%	97.21%

To further verify the effectiveness of the dual-branch ConvNeXt structure and determine whether the performance improvement mainly results from an increased number of parameters, comparative experiments with different fusion structures were designed. Three structures were compared:

1. Early fusion: GASF and GADF were directly used as a two-channel image input to a single ConvNeXt network at the input stage, and feature extraction and classification were performed by the same network.
2. Channel concatenation: Features were extracted from GASF and GADF separately and then concatenated along the channel dimension before being fed into the subsequent classification module.
3. Proposed dual-branch: Two ConvNeXt branches were used to perform decoupled modeling of GASF and GADF, respectively, and feature fusion was performed in the high-level feature space.

As shown in Table 7, different fusion strategies had a clear influence on diagnostic performance. Early fusion directly fused the GASF and GADF images at the input level and achieved an Accuracy of 94.17%. Channel concatenation extracted features from GASF and GADF separately and then concatenated them along the channel dimension, improving the Accuracy to 95.42%. This indicates that separate feature extraction helps preserve the distinct information contained in the two image representations.

**Table 7:** Comparison of different fusion strategies on the PU dataset.

Model	Fusion strategy	Parameters	Accuracy	Precision	Recall	F1-Score
Early fusion	Input-level fusion	1.58M	94.17%	94.32%	94.05%	94.18%
Channel concatenation	Channel concatenation fusion	2.59M	95.42%	95.56%	95.30%	95.43%
Proposed dual-branch	Dual-branch high-level fusion	2.53M	97.08%	97.34%	97.08%	97.21%

In contrast, the proposed dual-branch high-level fusion method achieved the best performance, with Accuracy, Precision, Recall, and F1-score values of 97.08%, 97.34%, 97.08%, and 97.21%, respectively. In addition, channel concatenation had more parameters than the proposed method, with 2.59M parameters compared with 2.53M, but achieved lower diagnostic performance. This result indicates that the performance improvement was not simply caused by an increased number of parameters, but was mainly attributed to the dual-view representation and high-level feature fusion strategy.

**Fig 7:**
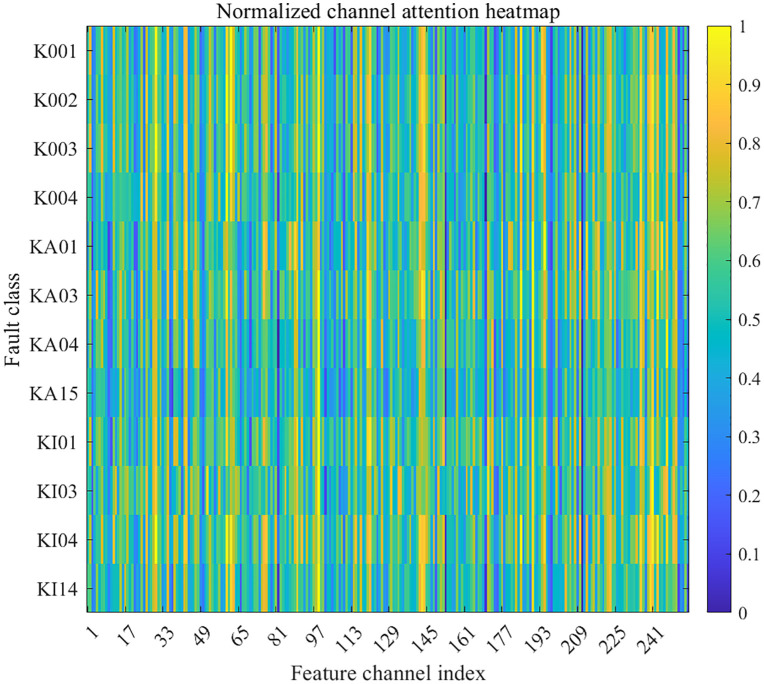
Normalized channel attention heatmap for different fault classes.

To analyze the feature attention mechanism of the CTransformer, the attention weights generated by the channel recalibration module were extracted, and the average channel weights for different fault categories were calculated. After normalization, the results were visualized in Fig 7. In this figure, the horizontal axis represents the deep feature channel index, the vertical axis represents the bearing health state, and the color indicates the normalized attention weight. It should be noted that these channels are not the original sensor channels, but deep feature channels obtained after GASF/GADF images were extracted and fused by the dual-branch ConvNeXt.

As shown in Fig 7, the distributions of high-weight channels vary across different fault categories, indicating that the model can adaptively focus on different fault-sensitive features according to the fault state. Some channels show strong responses across multiple categories, which may reflect common fault-related features, whereas other channels are more prominent only in specific categories and may be associated with local impacts, dynamic differences, or texture mutations. These results suggest that the channel attention mechanism helps improve both the discriminative capability and interpretability of the proposed model.

## Conclusion

To address the limited feature representation capability of one-dimensional vibration signals and the limitations of existing methods in multi-source feature fusion and global modeling for rotating machinery fault diagnosis, a fault diagnosis method based on GAF and dual-branch ConvNeXt-CTransformer was proposed. In the proposed method, raw vibration signals were first transformed into GASF and GADF image representations to enhance the structural representation of the data. Then, a dual-branch ConvNeXt network was constructed to extract the two types of features in parallel, enabling effective fusion of multi-source information. On this basis, a CTransformer was introduced to model global dependencies among the fused features and adaptively enhance key channel information, thereby improving the discriminative capability of the model.

Experimental results on the PU bearing dataset and the UC gear dataset showed that the proposed method outperformed the comparison methods in terms of accuracy, precision, recall, and F1-score, verifying its effectiveness and generalization capability. The ablation experiments further demonstrated the positive contribution of each key module to performance improvement.

Although promising results were achieved, some limitations remain in practical industrial applications. In future work, more robust feature modeling mechanisms will be investigated, and the applicability of the proposed method under cross-condition and real industrial scenarios will be further explored.
